# Voxelotor Treatment Providing Transfusion Independence for Patient With Combined Sickle Cell Disease and Lipopolysaccharide-Responsive and Beige-Like Anchor (LRBA) Deficiency

**DOI:** 10.7759/cureus.47144

**Published:** 2023-10-16

**Authors:** Kelsey M Larkin, Archana Sharma, Liz Castro, Richard Drachtman

**Affiliations:** 1 Pediatric Hematology, Robert Wood Johnson University Hospital, New Brunswick, USA

**Keywords:** autoimmune, hemoglobin, oxbryta, transfusion, hemolytic anemia, voxelotor, lrba deficiency, sickle cell disease (scd)

## Abstract

We present a 22-year-old female with transfusion-dependent anemia due to sickle cell disease (SCD) with lipopolysaccharide-responsive and beige-like anchor protein (LRBA) deficiency whose treatment frequency was moderated with voxelotor (Oxbryta®). The patient was transfusion dependent, initially thought to be secondary only to SCD. After the diagnosis of LRBA deficiency, her regimen included abatacept, sirolimus, hydroxyurea, and folic acid, but she still required intermittent transfusion. She was started on voxelotor in January 2020. Since initiation, her baseline hemoglobin level has increased and she is no longer transfusion dependent.

## Introduction

*LRBA *gene mutations have been linked to Common Variable Immune Deficiency 8 with autoimmunity, a rare autosomal recessive disease, with a < 1/1,000,000 prevalence caused by bi-allelic mutations in the *LRBA *gene [1, 2]. These mutations can be distributed throughout the gene and include missense, splice sites, small indels, and nonsense mutations [3]. It commonly presents as early-onset recurrent infections and autoimmune disorders, including immune thrombocytopenic purpura, autoimmune hemolytic anemia (AIHA), and/or inflammatory bowel disease [1]. Other potential manifestations include lymphoproliferative disease, autoimmune enteropathy, thyroiditis, dermatitis, type 1 diabetes, and inflammatory arthritis. An Iranian study found that autoimmune cytopenia was the most common autoimmune manifestation [4].

Mutations prevent normal production of the LRBA protein, a cytosolic protein that maintains intracellular stores of cytotoxic T lymphocyte-associated protein (CTLA-4) by binding the cytoplasmic tail and preventing lysosomal degradation. CTLA-4 binds CD-80/86, transmitting the inhibitory signal that downregulates T-cell immune response to maintain peripheral tolerance. Depletion of CTLA-4, as seen in LRBA deficiency, leads to abnormal T-cell activity. The symptoms of LRBA deficiency are similar to those of CTLA-4 deficiency; however, LRBA deficiency leads to much lower levels of CTLA-4, explaining the earlier onset of disease, higher severity, and almost complete incidence of symptoms.

The pathogenesis of AIHA, although not completely understood, is known to be mediated by autoantibodies against erythrocytes, leading to premature destruction of red blood cells. Dysregulation of both B and T cells and abnormal red blood cell membrane structures lead to autohemolysis. In the case of LRBA deficiency, dysregulation of immune self-tolerance due to uncontrolled T-cell activity causes widespread autohemolysis as autoantibodies interact with antigens on the surface of the erythrocytes. Conditions that cause red blood cell membrane deformities, additionally mark cells for autohemolysis. In the case of SCD, a single-point mutation alters the β-globin gene, rendering the red blood cells susceptible to polymerization when deoxygenated. The sickled red blood cells get trapped in microvessels, leading to early removal via hemolysis. Additionally, sickled erythrocytes adhere to endothelial cells, platelets, and polymorphonuclear neutrophils, initiating an inflammatory cascade that releases reactive oxygen species that independently trigger hemolysis [5].

Patients with LRBA deficiency have traditionally been treated with abatacept (Orencia®), a fully human recombinant protein comprising the extracellular domain of CTLA-4 and the Fc portion of IgG. It functions by binding CD-80/86 and inhibiting T-cell activation [6,7]. Additionally, patients are treated with sirolimus (Rapamune®), an mTOR (mammalian target of rapamycin) inhibitor that binds FKBP (FK506-binding protein) and inhibits IL-2 response, preventing T-cell activation and B-cell differentiation [8]. A recent international retrospective study observed outcomes for patients with LRBA mutations receiving hematopoietic stem cell transplants. In successful transplants, patients had less immune dysregulation than in conventional treatment with immunosuppressive agents, including corticosteroids, sirolimus, and abatacept [9].

## Case presentation

We present a 22-year-old female with severe, transfusion-dependent anemia due to sickle cell disease (SCD) (homozygous Hb SS) with LRBA deficiency whose treatment frequency was moderated with voxelotor (Oxbryta). The patient was diagnosed with SCD on newborn screening and her early course had been complicated by acute chest syndrome and pain crises treated with hydroxyurea. In 2015, she developed severe anemia refractory to increased hydroxyurea dosages. In December 2015, she had intermittent and progressive eye and joint swelling and pain and developed marked lymphadenopathy in early March 2016. A lymph node biopsy revealed lymphoproliferation but was EBV (-) and not malignant, suggesting a potential autoimmune etiology. Rheumatologic workup revealed elevated IgG and was Coombs (+), Rh (+), and ANA (-).

In March 2016, she was admitted for severe acute hemolytic anemia with a hemoglobin (Hb) of 5.1 g/dL and reticulocyte count of 21%. At that time, biopsy of a maculopapular rash (which first appeared in 2014) on the bilateral lower extremities revealed cutaneous leukocytoclastic vasculitis. No further systemic vasculitis was found and cryoglobulins were negative. She was treated with prednisone and 4 weekly doses of rituximab. In May 2016, she presented with a Hb level of 5.4 g/dL and reticulocyte count of 28%.

During this admission, a transfusion medicine specialist was consulted to determine the etiology of her persistent hemolysis. It was suggested this was not AIHA because the patient’s direct Coombs test (DAT) at this time was negative. While the etiology was still unclear, they determined she could be having a hyperhemolytic crisis, where, for an unknown reason, both the transfused and the patient’s own red blood cells are destroyed. For this reason, it was determined that transfusion would offer minimal reprieve and should be avoided. Alternate treatment with intravenous immunoglobulin (IVIG) was initiated, but hemoglobin still dropped to 4.7 g/dL with reticulocyte count of 52%. The patient again received a transfusion and was discharged home with Hb level of 9.6 g/dL and reticulocyte count of 10.4%.

A week later, she presented with severe, generalized body pain and was given another dose of IVIG and increased prednisone, but Hb dropped to 4.6 g/dL with a reticulocyte count of 34%. Rituximab treatment was initiated with 4 weekly doses and prednisone was increased until levels stabilized. In July 2016, the patient was admitted due to pain and significant hemolysis. The decision was made to begin mycophenolate mofetil, receive a dose of IVIG, and increase hydroxyurea.

The patient was referred to NIH in November 2016 for genetic sequencing given the unclear etiology of ongoing hemolysis and mutation analysis confirmed a defect in the LRBA gene. Based on NIH recommendations, on December 13, 2016, the patient began treatment with abatacept and currently remains on monthly infusions. In April 2017, the patient began prednisone taper from 10 mg dosage, which was well tolerated with one drop in hemoglobin during the 2.5 mg decrease every 2 weeks.

Nine months after initiation of LRBA deficiency treatment, the patient presented with a vaso-oclusive crisis, fever, and an acute drop in hemoglobin. The patient was parvovirus B-19 IgM-positive and received high-dose prednisone and two doses of IVIG preceding an additional transfusion. The patient was discharged at a stable Hb level of 7 g/dL and 1.5% reticulocyte count. Following NIH recommendation, the patient began sirolimus treatment in September 2017, in addition to the abatacept initiated in December 2016. Hemoglobin levels were improving (6.9 g/dL), but there was a sharp increase in reticulocyte count (45%) that warranted a dose of IVIG in October 2017.

Despite maximal doses of abatacept and sirolimus, the patient continued to experience episodes of vaso-occlusive pain crises and drops in hemoglobin. The patient reported lapses in medication adherence due to social barriers and difficulty remembering to take medications. After establishing support, the patient remained compliant. Voxelotor was initiated in January 2020 after the FDA accelerated approval. Since initiation, the patient’s baseline hemoglobin level has seen significant improvement (Table 1) and has not required transfusion. The patient’s current regimen for treatment of SCD and hemolytic anemia is abatacept 720 mg monthly; sirolimus 3 mg BID; voxelotor 1,000 mg QD; hydroxyurea 2,000 mg QD; and folic acid 1 mg QD.

**Table 1:** Hemoglobin levels throughout the treatment course Bold values indicate levels after voxelotor initiation. Reference value for female hemoglobin level: 12.1 - 15.1 g/dL.

Date	Hemoglobin Level
1/17/2016	5.8 g/dL
5/5/2016	5.4 g/dL
1/27/2017	6.1 g/dL
10/27/2017	6.9 g/dL
4/11/2018	6.1 g/dL
5/10/2019	5.6 g/dL
11/27/2019	6.8 g/dL
3/24/2020	6.9 g/dL
10/2/2020	8.1 g/dL
6/3/2021	8.5 g/dL
7/22/2022	8.9 g/dL
9/29/2022	8.4 g/dL

## Discussion

Voxelotor, a novel HbS polymerization inhibitor, was granted accelerated approval to treat SCD and associated complications in 2019 [10]. It functions by binding reversibly to Hb and increasing affinity for oxygen. This prevents polymerization and sickling, which minimizes blood viscosity and improves blood cell deformability - reducing episodes of hemolysis and improving anemia [11]. A phase 3 randomized, double-blind, placebo-controlled study evaluating the safety and efficacy of voxelotor treatment showed a mean change in hemoglobin from baseline of 1.1 g/dL compared with −0.1 for placebo (P < 0.001). The efficacy of this medication was significant and since approval has been implemented in the treatment regimens for many patients with SCD. The impact on hemoglobin levels may prove to be life-changing for many [12].

The patient presented here was transfusion-dependent as a result of both her LRBA deficiency and SCD. Despite maximal dosages of abatacept and sirolimus, she was unable to maintain an appropriate Hb level. Since starting voxelotor in January 2020, she has had significantly less admissions and maintains a more stable hemoglobin level (Table 1). There have been no adverse effects attributable to the addition of voxelotor to her medication regimen. Given the rapid success of this medication for patients with complicated medical diagnoses, further consideration should be given to other SCD cases presenting with concurrent diagnoses and refractory symptoms. LRBA deficiency is a rare genetic disorder that lacks a well-defined treatment plan. Our patient’s deficiency manifests most profoundly as hemolytic anemia, which resulted in a difficult-to-maintain hemoglobin level. Due to the mechanism of action of voxelotor, it may be a novel agent for diseases in which hemolytic anemia may occur.

There are no other reported cases of patients with co-existing SCD and LRBA deficiency that have been treated with voxelotor in the literature. Our patient had a complicated and transfusion-dependent course of hemolytic anemia, requiring NIH genetic sequencing. After diagnosis of LRBA deficiency, more could be understood about the underlying mechanism of the patient's clinical presentation. With more understanding, treatment options could be explored and trialing voxelotor showed a substantial improvement in hemoglobin stability and clinical condition. Diagnosis of LRBA deficiency allowed for more expansive treatment options and therefore LRBA deficiency and treatment may be considered for patients with refractory hemolytic anemias.

## Conclusions

This is the first reported case of a patient with co-existing SCD and LRBA deficiency, which has caused severe, transfusion-dependent hemolytic anemia. After extensive genetic testing at NIH, an official diagnosis was established and subsequent treatment with voxelotor led to stabilization of hemoglobin levels with a substantial reduction in transfusion requirements. In conclusion, LRBA deficiency should be considered in patients with refractory hemolytic anemia, and effective treatment regimens may be established.
